# Prediction of breast self-examination in a sample of Iranian women: an application of the Health Belief Model

**DOI:** 10.1186/1472-6874-9-37

**Published:** 2009-12-29

**Authors:** Sedigheh Sadat Tavafian, Laleh Hasani, Teamur Aghamolaei, Shahram Zare, David Gregory

**Affiliations:** 1 Department of Health Education, Faculty of Medical Sciences, Tarbiat Modaress University, Tehran, Iran; 2 Department of Mental Health, Iranian Institute of Health Sciences Research, Tehran, Iran; 3 Department of Public Health, School of Health, Hormozgan University of Medical Sciences, Bandar Abbas, Iran; 4 Department of Social Medicine, School of Medicine, Hormozgan University of Medical Sciences, Bandar Abbas, Iran; 5 Faculty of Health Sciences, University of Lethbridge, Lethbridge, Alberta, Canada

## Abstract

**Background:**

Iranian women, many of whom live in small cities, have limited access to mammography and clinical breast examinations. Thus, breast self examination (BSE) becomes an important and necessary approach to detecting this disease in its early stages in order to limit its resultant morbidity and mortality. This study examined constructs arising from the Health Belief Model as predictors of breast self examination behavior in a sample of women living in Bandar Abbas, Iran.

**Methods:**

This study was conducted in eight health centers located in Bandar Abbas, Iran. The sample consisted of 240 eligible women who were selected from referrals to the centers. The inclusion criteria were as follows: aged 30 years and over; and able to read and write Farsi. Women with breast cancer, who were pregnant, or breast feeding, were excluded from the study. Data were collected by using a self administered questionnaire which included demographic characteristics and Champion's Health Belief Model Scale. This instrument measures the concepts of disease susceptibility (3 items), seriousness (6 items), benefits (4 items), barriers (8 items) and self-efficacy (10 items).

**Results:**

The subjects' mean age was 37.2 (SD = 6.1) years. Just under a third of the subjects (31.7%) had performed BSE in the past and 7.1% of them performed it at least monthly. Perceived benefits and perceived self-efficacy of the women who performed BSE were significantly higher compared with women who did not practice BSE (p < 0.03). Furthermore, perceived barriers were lower among those who had performed BSE (p < 0.001). Logistic regression analysis indicated that women who perceived fewer barriers (OR: 0.70, 95% CI: 0.63-0.77, p < 0.001) and had higher self-efficacy (OR: 1.08, 95% CI: 1.02-1.13, p = 0.003) were more likely to perform BSE (R^2 ^= 0.52).

**Conclusion:**

Findings from this study indicated that perceived barriers and perceived self-efficacy could be predictors of BSE behavior among the sample of women. Therefore, BSE training programs that emphasize self-efficacy and address perceived barriers are recommended.

## Background

Breast cancer is the most common cancer [1,2] and contributes to a high rate of death among women worldwide [3,4]. It has been estimated that one out of every nine women living in western countries is likely to be afflicted by breast cancer in her lifetime [4]. The incidence of breast cancer varies between countries; the highest rates occur in the United States and Canada and, the lowest rate is found in Asia. The Nordic countries have recently reported a steady increase in the incidence of breast cancer. A high prevalence rate of breast cancer is noted among women living in Denmark, Finland and Sweden [5]. The incidence rate of breast cancer among Asian women has also increased in recent years and is likely related to life style changes [6]. In Egypt, breast cancer is the most frequently diagnosed cancer among women, and it comprises 25.5% of all cancers in that country [7]. Breast cancer is the second leading cause of cancer death and accounts for 24% of female cancers in Turkey [8].

In Iran, cancer is the third cause of death [9], and breast cancer is the second most common cancer among women [10]. Although breast cancer prevalence/incidence data are limited to non-existent within the Health Ministry of Iran, it is likely that the incidence rate of the disease is increasing. Furthermore, Iranian women are most often diagnosed with advanced breast cancer (i.e., Stage III and IV) and they are relatively younger than their western counterparts [11].

One study conducted in Iran [10] revealed that the incidence rate of breast cancer was 17.09 per 100,000 women with a mean age of 51.3 years (SD = 12.5). However, a literature review of articles from January 1998 to December 2005 found that the incidence rate of breast cancer among Iranian women was 22 per 100,000, while the prevalence rate was determined as 120 per 100,000[12]. This study [12] indicated that eighteen percent of breast cancers in Iranian women were diagnosed as Stage 1, 57% as Stage 2, and 25% as Stage 3. Most women (72%) were diagnosed with a tumor over 2 cm and 63% of them had lymph node involvement at the time of diagnosis [12]. In a recent study, the incidence rate of breast cancer in Iran was reported as 17.44/100,000 population in 2005-2006. The highest rate of breast cancer (69.28 per 100,000 population) occurred in the 55-59 age group, while the lowest rate (0.02 per 100,000 population) was evident among women aged 15 to 19 years [13]. The researchers established that the warning signs of breast cancer (eg., painless lump, nipple retraction, bloody discharge from the nipple) were not well known among Iranian women. Furthermore, they concluded that BSE, mammography, and clinical breast examinations were inadequate in terms of their practice and availability [13].

Early detection of breast cancer plays an important role in reducing its morbidity and mortality. Theoretically, a 95% survival rate could be achieved if this cancer was diagnosed at an early stage [3]. BSE, mammography, and clinical breast examination are considered as screening methods for early detection breast cancer [14]. Although there is controversy surrounding the efficacy of BSE in countries where mammography and clinical breast exams are readily available [15], elsewhere BSE remains a cost-effective method to detect breast cancer. A woman who performs regular BSE may be more motivated to seek medical attention, including mammography and clinical breast exams if available [15,16]. Given that Iranian breast cancer patients are relatively younger than their counterparts in western countries, breast cancer screening programs should be accorded more attention by public health professionals in Iran [11].

Despite the relative benefits of BSE, its application remains low [17]. Studies conducted among different groups of women in United States, showed that monthly BSE rates ranged from 29% to 63% [18,19]. A study conducted in Nigeria revealed that only 18.1% of participants reported regular application of BSE [20]. Similar results were found among Iranian women with only 17% conducting regular BSE. The researchers concluded that Iranian women did not know how to perform a BSE [13]. Variables such as demographic characteristics, knowledge, and education influence the practice of BSE [20-23]. Furthermore, a lack of belief regarding the necessity of regular BSE has an impact on the engagement of this screening behavior [14-16,19]. Understanding women's beliefs regarding BSE can be used to design appropriate educational interventions which promote this screening behavior [3,14-16,19,24].

The Health Belief Model (HBM) is a psychosocial model that accounts for health behaviors by identifying factors associated with individuals' beliefs which influence their behaviors [24]. According to this model, individuals who perceive themselves as susceptible to a certain disease (perceived susceptibility), who perceive that the disease has potentially serious consequences (perceived severity), who believe that preventive actions will cause positive outcomes (perceived benefits), who perceive that barriers to taking preventive actions are outweighed by the benefits, and who believe that they are able to engage in a certain preventive health behavior (self-efficacy), are more likely to engage in that health behavior [3]. This model has been widely used to examine beliefs related to breast cancer screening behaviors such as BSE [14-18].

To the best of our knowledge, there is no previous research which that applied the HBM to understand BSE beliefs and associated factors among Iranian women [6,11,21,25,26]. Therefore in this study, BSE predictive factors arising from HBM were examined in a sample of Iranian women. This study differs from previous Iranian researches due to applying HBM to predict BSE. In addition, for the first time, in this study Champion's revised Health Belief Model Scale (CHBMS) was used to collect data.

## Methods

This was a cross sectional study conducted in health centers of Bandar Abbas from September to November 2008. There were no ethical issues encountered during the course of this study. The study was approved by the Medical Ethics Committee of Hormozgan University of Medical Sciences.

### Sampling procedure

Bandar Abbas, a city in south of Iran, has eight health centers in which primary health services such as family planning, immunization, and child development assessment are offered to healthy women or their children. Since there were eight health centers in Bandar Abbas and the city's population was equally dispersed around each center, the sample size of each center was the same. Among women referred to these eight health centers for health services, 30 eligible subjects who were older than 30 years and able to read and write Farsi were recruited from each center. Thus a total of 240 women participated in the study. Women who were previously diagnosed with breast cancer, who were pregnant or who were breast feeding at the time of recruitment were excluded from the study. In terms of the sampling frame, we identified the first referral if the woman was eligible and agreed to take part in the study. The second prospective subject was the 10^th ^referral if she was eligible and if she consented to participate in the study. If this subject did not meet the sampling criteria or if she refused to participate, then the next referral (11^th^) was selected and so on. Thus every other 10 women were recruited until 30 subjects were attained from each of the eight health centers. The final sample size was similar to previous studies which have made use of Champion's revised Health Belief Model Scale [15-17].

### Measures

A self administered questionnaire capturing socio-demographic characteristics and Champion's revised Health Belief Model Scale (CHBMS) were used as data collection instruments for this study. Socio-demographic characteristics included age, level of education, and marital status. In this study, BSE was the dependent variable. In addition to the CHBMS, subjects were asked two questions about performing BSE. (1) Do you perform breast self examination? (2) If yes, how often do you perform it? if the response was positive the second question was asked. BSE was considered "regular" if it had been performed at least once a month and irregular if it had not been monthly.

With the exception of age and BSE frequency, all questions in the study were in a Likert format. The CHBMS was developed in 1984 and it has been revised three times [3,24,27,28]. It is a commonly used instrument to measure the Health Belief Model (HBM) variables including susceptibility, seriousness, perceived benefits, perceived barriers, self-efficacy and health motivation associated with breast cancer screening [24]. The original scales were tested and found to be valid and reliable for measuring BSE practice and breast cancer beliefs [24,28,29].

The latest version of the scale was adapted for the present study to measure all sub-scales except health motivation [24]. The questionnaire consisted of 31 items. All items offered five response choices ranging from "strongly disagree (scores 1 point)" to "strongly agree (scores 5 points)". Higher scores indicated a positive attitude towards BSE except for barriers to BSE. Susceptibility of breast cancer consisted of three items scored from 3 to 15, seriousness of breast cancer consisted of six items scored from 6 to 30, BSE benefits consisted of four items scored from 4 to 20, BSE barriers consisted of eight items scored from 8 to 40 and BSE self-efficacy consisted of 10 items scored from 10 to 50. Motivation for BSE was not assessed in this study.

Prior to data collection, the CHBMS was translated in Persian using a backward-forward translation technique. To do this, a panel of experts translated the CHBMS items from English to Farsi language and then it was back-translated into English. Minor translation adjustments were carried out until the two versions (Farsi/English formats) were identical. The reliability coefficient for each subscale was calculated using Cronbach's alpha. Cronbach's alpha coefficients for the original CHBMS for susceptibility, seriousness, BSE benefits, BSE barriers and BSE self-efficacy were 0.87, 0.80, 0.69, 0.83 and 0.90 respectively [30]. In the study by Gozum and Aydin, Cronbach's alpha coefficients for susceptibility, seriousness, BSE benefits, BSE barriers and BSE self-efficacy were 0.69, 0.75, 0.83, 0.73 and 0.82 respectively [15]. In our study, Cronbach's alpha coefficients for susceptibility, seriousness, BSE benefits, BSE barriers and BSE self-efficacy were calculated as 0.68, 0.77, 0.78, 0.77 and 0.87 respectively. With the exception of susceptibility, all coefficients were between 0.7 and 0.9.

Data were collected through self administrated questionnaires. Before any data were obtained, subjects were informed about the risks and benefits of the study and written consent was obtained from each subject. None of the women refused to participate. To ensure confidentiality, women were asked not to provide their names.

### Data analysis

Data were analyzed using SPSS13 software and p < 0.05 was considered statistically significant.164 women reported never performing BSE and 76 reported having performed it in the past, among them 17 participants reported performing BSE at least monthly. Then, to analyzing, sample was divided into two parts according to weather or not they had performed BSE at all. Independent t-tests was used to compare health belief model constructs between women who performed BSE (n = 76), and who did not perform it (n = 164). Logistic regression analysis was used to predict the probability that women would or would not perform BSE in the future.

## Results

In total, 240 women participated in the study. The subjects' mean age was 37.2 years (SD = 6.1) and most (95.8%) were married. Just under a third of the subjects (31.7%) had performed BSE in the past and 7.1% of them performed it at least monthly. Table 1 shows the demographic characteristics for the sample. There were no statistical differences in BSE performance with respect to age, level of education, or history of breast problems (p > 0.05). Factors arising from the HBM were compared for BSE performers (regular and irregular) and non-performers (never done) in Table 2. As the data in this table indicate, perceived BSE benefits and self-efficacy among subjects who performed BSE (regularly and irregularly) were significantly higher than those who never performed it (p < 0.05). However, perceived BSE barriers among BSE performers (regular and irregular) were significantly lower than those of non performers (p < 0.05). Table 3 shows the comparison of health belief model factors between women who reported practicing BSE and those who never performed it. There were significant differences between these two groups of women with respect to the HBM factors mostly related to self-efficacy (8 items).

**Table 1:** Demographic characteristics of the sample

Variable	Mean (SD)	N	%
**Age**	37.2 (6.1)		
**Marital status**			
Married		231	96.3
Widowed/Divorced		9	3.8
**Education**			
Primary		70	29.2
Secondary		52	21.6
High school		78	32.5
University		40	16.7
**History of breast problems**			
Yes		29	12.1
No		211	87.9
**Performing BSE**			
Yes (regular* & irregular**)		76	31.7
No (never done)		164	68.3

*BSE has been considered "regular" if it had been done monthly.

** BSE has been considered irregular if it had not been done monthly.

**Table 2:** Comparison of Health Belief Model factors among performers and non-performers of BSE

	Performing BSE				
Sub-scales	Yes (regular* & irregular**) (n = 76)		*No (never done) *(n = 164)	t	p*
	Score range	Mean (SD)	Mean (SD)		
Susceptibility	3-15	7.68(2.5)	7.67(2.4)	0.23	0.97
Seriousness	6-30	21.10(5.6)	21.80(4.7)	0.53	0.27
BSE benefits	4-20	17.34(2.7)	16.53(3.1)	1.80	0.03
BSE barriers	8-40	16.36(5.3)	19.55(5.7)	-10.7	<0.001
BSE self-efficacy	10-50	35.90(7.6)	28.30(7.5)	4.10	<0.001

*Independent t-test

**Table 3:** Comparison of Health Belief Model items among performers and non-performers of BSE (Score range: 1-5)

	*Performing BSE*			
Item	*Yes (regular/irregular)*	*No (never done)*	t*	p
	Mean (SD)	Mean (SD)		
**Susceptibility**				
1. It is likely that I will get breast cancer	2.63(1.1)	2.45(1.02)	1.24	0.21
2. My chances of getting breast cancer in the next few years are great	2.76(1.03)	2.73(1.05)	0.17	0.86
3. I feel I will get breast cancer sometime during my life	2.41(1.07)	2.43(1.04)	-0.17	0.86
**Seriousness**				
4. The thought of breast cancer scares me	3.86(1.2)	3.81(1.25)	0.29	0.76
5. When I think about breast cancer, my heart beats faster	3.85(1.3)	3.68(1.2)	0.96	0.34
6. I am afraid to think about breast cancer	3.75(1.3)	3.77(1.2)	-0.14	0.88
7. Problems I would experience with breast cancer would last a long time	3.41(1.2)	3.33(1.2)	0.43	0.67
8. Breast cancer would threaten a relationship with my husband	3.06(1.4)	3.17(1.2)	-0.58	0.56
9. If I had breast cancer my whole life would change	3.64(1.3)	3.63(1.2)	0.06	0.95
**Benefit BSE**				
10. When I do BSE, I am doing something to take care of myself	4.30(0.9)	4.17(1.01)	0.92	0.35
11. Completing BSE each month may help me find breast lumps early	4.42(0.78)	4.26(0.87)	1.30	0.19
12. Regular BSE decreases the rate of death from breast cancer	4.43(0.75)	4.18(0.96)	2.01	0.04**
13. If I find a lump early through BSE, my treatment for breast cancer may not be as bad	4.23(1.01)	4.04(1.04)	1.35	0.17
				
**Barrier BSE**				
14. BSE is embarrassing to me	1.69(0.93)	2.15(1.18)	-2.98	0.003**
15. BSE takes too much time	1.65(0.88)	2.08(1.07)	-3.03	0.003**
16. It is hard to remember to do breast examination	1.97(1.15)	2.45(1.18)	-2.95	0.003**
17. I don't have enough privacy to do breast examination	1.63(0.87)	2.20(1.17)	-3.77	<0.001**
18. BSE is not necessary if you have a breast exam by a healthcare provider	2.89(1.5)	2.87(1.3)	0.08	0.93
19. BSE is not necessary if you have a routine mammogram	3.02(1.4)	2.95(1.3)	0.36	0.71
20. My breast too large for me to complete BSE	1.80(0.96)	1.94(0.93)	-1.08	0.27
21. I have other problems more important than doing BSE	1.88(1.1)	2.15(1.1)	-1.75	0.08
**BSE self-efficacy**				
22. I know how to perform BSE	3.68(1.1)	3.06(1.2)	3.67	<0.001**
23. I can perform BSE correctly	3.50(1.2)	3.00(1.2)	2.93	0.004**
24. I could find a breast lump by performing BSE	3.73(1.05)	3.14(1.1)	3.72	<0.001**
25. I am able to find a breast lump that is the size of a walnut	3.90(1.2)	3.34(1.2)	3.35	<0.001**
26. I am able to find a breast lump that is the size of a hazelnut	3.57(1.2)	3.05(1.2)	3.11	0.002**
27. I am able to find a breast lump that is the size of a pea	2.75(1.3)	2.68(1.2)	0.34	0.73
28. I am sure of the steps to follow for doing BSE	3.45(1.1)	2.91(1.2)	3.07	0.002**
29. I am able to tell something is wrong with my breast when doing BSE	3.84(1.02)	3.18(1.2)	4.05	<0.001**
30. I am able to tell something is wrong with my breast when I look in the mirror	2.84(1.2)	2.77(1.2)	0.39	0.69
31. I can use the correct part of my fingers when examining my breasts	3.61(1.1)	3.07(1.2)	3.17	0.002**

*t-test **significant

Logistic regression analysis was used to predict the probability of performing BSE. Health belief model factors (susceptibility, seriousness, BSE benefits, BSE barriers and BSE self-efficacy) were entered into the logistic regression analysis - as independent variables - to be tested as predictor factors for BSE performance. Table 4 shows the results of the logistic regression analysis. The women who perceived more self-efficacy (OR = 1.08) and fewer barriers (OR = 0.70) were more likely to perform BSE. One increase in each BSE self-efficacy score made the chances of BSE 1.08 times more likely. Similarly one decrease in BSE barrier scores made the probability of BSE increased up to 30%. The logistic regression model correctly classified 90.9% of the BSE non performers and 60.5% of the performers, with the overall rate of 81.3%, compared with expected chance of 50%.

**Table 4:** Logistic regression analysis of Health Belief Model factors for predicting BSE behavior

Variable	B	S.E	Wald	Odds ratio	CI*	p
Susceptibility	0.004	0.075	0.003	1.01	0.86-1.16	0.95
Seriousness	0.052	0.036	2.092	1.05	0.98-1.13	0.14
BSE benefits	-0.138	0.077	3.236	0.87	0.75-1.01	0.07
BSE barriers	-0.355	0.050	50.183	0.70	0.63-0.77	<0.001
BSE self-efficacy	0.075	0.025	8.881	1.08	1.02-1.13	0.003
Constant	3.897	1.933	4.063	49.2	-----------	0.05

Model chi-square = 111.2, df = 5, p = < 0.001 Nagelkerke R Square = 0.52

*Confidence interval for odds ratio

## Discussion

Breast cancer is one of the most frequent cancers occurring among Iranian women. Delay in diagnosis and treatment of this disease decreases survival rates [21]. Within the Iranian context, breast self examination provides a reasonable screening method for early detection of treatable breast tumors [20]. Recent evidence challenges the efficacy of BSE for women aged 30 years and over [31], and western women are not encouraged to conduct regular BSE. However, Iranian women need to exercise some sense of control over breast cancer, especially given the limited availability of mammograms and clinical breast exams compared to western countries. Thus, it is not unreasonable to empower Iranian women and encourage them to conduct BSE.

This study revealed that few participants performed BES on the regular basis. In consistent with our study, previous research suggests that in Iran, the rate of BSE is not satisfactory [6,13,32]. These researches established that the low rate of BSE among Iranian women was related to socio-economic status [13,25], lower levels of education [21,25], lack of breast cancer knowledge and lack of knowledge regarding the conduct of BSE [13,21,25,32], and negative family history of breast cancer [32]. However, there are disparate findings concerning factors that impact BSE, like a study established that personal experience or a family history of breast cancer were not related to performing BSE [13]. Furthermore, a study of Malaysian teachers identified that there was no association between socio demographic characteristics such as age, marital status, and family history of breast cancer and BSE behavior. Rather, variables such as breast cancer knowledge, awareness of breast cancer screening methods, and regular visits with a physician influenced BSE behavior [22]. Of interest, in developed countries, there are higher rates of regular BSE [17-19]. Thus, the contexts in which women live likely affect those factors which impact the extent to which BSE is practiced.

The majority of the respondents in this study believed that their relative risk of breast cancer was the same as other women. However, only 6% of them reported performing BSE on a regular basis. Furthermore, the women, who did not perform BSE, believed that it was not necessary [6]. This finding was also supported in another Iranian study [32]. Beliefs and behaviors of Iranian teachers toward early detection of breast cancer and BSE were reported by Jarvandy in 2002 [6]. Health beliefs related to BSE have been documented in a sample of Turkish women who are similar to Iranian women in terms of socio-cultural characteristics. In this Turkish study, women who were more confident in their abilities to perform BSE were more likely to engage in this screening behavior [8]. As the existing knowledge base regarding Iranian women's attitudes and beliefs towards BSE is limited, further research in this area is strongly recommended.

The results of this current study showed that subjects who regularly performed BSE, perceived more BSE benefits, fewer BSE barriers and more BSE self-efficacy than those women who had never performed this behavior. These findings are in alignment with the constructs of HBM that predicts women who perceive themselves to be susceptible to breast cancer (perceived susceptibility) and who also believe that breast cancer is a serious disease (perceived seriousness) are more likely to practice regular BSE. Contrary to the HBM, the present study did not establish any associations between perceived susceptibility/seriousness and BSE. One explanation of this finding may be related to the inadequate knowledge of Iranian women regarding seriousness of breast cancer [14]. However, knowledge regarding breast cancer was not established and is considered a limitation of the study. Future studies that examine the relationship between perceived susceptibility/seriousness and BSE among Iranian women are recommended.

The women in the study identified barriers that undermined their abilities to practice BSE on a regular basis. For the subjects who did not perform BSE, they observed that BSE was an embarrassing behavior and that they did not have enough privacy to engage in this practice. Some of these women also suggested that regular BSE required too much time; for others it was a matter of trying to remember to do BSE regularly. Health program planners would be wise to consider these barriers in designing effective interventions to improve BSE.

Although perceived benefits were associated with BSE in univariate analysis, it was not a predictor of BSE according logistic regression analysis. This result is in contrast to what was reported in a study among Turkish women regarding perceived benefits; this study concluded that women who perceived more benefits in relation breast self examination, were more likely to engage in the behavior [15]. Women who were more confident in their ability to conduct BSE-subjects with higher self efficacy scores-were more likely to perform BSE. This finding was supported by previous research [17,33-35]. The findings also showed that a lack of skill in the performance of BSE was associated with limited to no BSE activity. Therefore, educational interventions that foster BSE skills and efficacy would likely contribute to higher rates of its performance.

## Conclusion

The findings of this study indicated that perceived BSE barriers and perceived BSE self-efficacy were factors predictive of BSE behavior among a sample of Iranian women. Therefore, BSE training programs that improve self efficacy and reduce or reframe barriers to BSE are strongly recommended.

## List of abbreviations Used

BSE: Breast Self Examination; HBM: Health Belief Model; CHBMS: Champion's revised Health Belief Model Scale.

## Competing interests

The authors declare that they have no competing interests.

## Authors' contributions

SST contributed to analyze the data and helped to draft the manuscript. LH participated in data collection and conducted the study. TA designed the study and drafted the manuscript. SZ performed statistical analysis. DG assisted with drafting the manuscript and edited it. These authors contributed equally to this work. All authors have read and approved the final version of the manuscript.

## Pre-publication history

The pre-publication history for this paper can be accessed here:

http://www.biomedcentral.com/1472-6874/9/37/prepub
